# High-Intensity Ultrasound Processing of *Aloe vera* (*Aloe barbadensis* Miller): Effect on Rheology, Phenolic Compounds, and Antioxidant Activity

**DOI:** 10.3390/foods15142414

**Published:** 2026-07-08

**Authors:** María de los Ángeles Sáenz-Esqueda, Juan José Martínez-García, María Mota-Ituarte, Jesús Josafath Quezada-Rivera, Armando Quintero-Ramos, María José Rivas-Arreola, Antoni Femenia, Rafael Minjares-Fuentes

**Affiliations:** 1 Facultad de Ciencias Químicas, Universidad Juárez del Estado de Durango, Gómez Palacio 35010, Mexico; angelesujed@ujed.mx (M.d.l.Á.S.-E.); juanjose.martinez@ujed.mx (J.J.M.-G.); mariamotaituarte@gmail.com (M.M.-I.); 2 Facultad de Ciencias Biológicas, Universidad Juárez del Estado de Durango, Gómez Palacio 35010, Mexico; josafath.quezada@ujed.mx; 3 Facultad de Ciencias Químicas, Universidad Autónoma de Chihuahua, Circuito Universitario, Campus II, Chihuahua 31125, Mexico; aquinter@uach.mx; 4 Science and Engineering Department, Universidad Iberoamericana Puebla, San Andres Cholula 72810, Mexico; mariajose.rivas@iberopuebla.mx; 5 Department of Chemistry, University of the Balearic Islands, Ctra Valldemossa Km 7.5, 07122 Palma de Mallorca, Spain; antoni.femenia@uib.es

**Keywords:** *Aloe vera*, high-intensity ultrasound, rheology, aloin, phenolic compounds, antioxidant capacity

## Abstract

High-intensity ultrasound (HIUS) is a non-thermal processing technology with the potential to modify the functionality of plant-derived materials. This study evaluated the effect of HIUS on the techno-functional properties, rheology, phenolic profile, aloin content, and antioxidant activity of *Aloe vera* gel at 11, 28, and 43 W/cm^2^ for 2.5, 5, and 7.5 min. HIUS reduced swelling capacity from 284.92 mL/g in the untreated sample by up to ~60%, while water retention capacity increased from 45.62 g/g to values close to 90 g/g. Fat adsorption capacity reached its highest value at 28 W/cm^2^ for 5 min (~60 g/g). Rheological analysis confirmed shear-thinning behavior and a marked viscosity reduction after sonication, with zero-shear viscosity ranging from 0.055 to 0.569 Pa·s. Total phenolic content decreased from ~6.0 mg GAE/g dm in the untreated gel to 2.6–3.3 mg GAE/g dm after HIUS. Aloin showed a non-linear response, increasing from ~43 to ~48 mg/g at 28 W/cm^2^ when processing time increased from 2.5 to 5 min, followed by an approximately 20% decrease at 7.5 min. Antioxidant activity ranged from 31 to 47% DPPH inhibition and 75 to 150 µmol TE/g ORAC. These findings indicate that moderate HIUS conditions improve selected functional properties while limiting bioactive compound degradation.

## 1. Introduction

The efficient utilization and valorization of agro-food resources have become central challenges in the transition toward more sustainable and circular food systems. In this context, the development of innovative processing strategies capable of enhancing the functional and bioactive properties of plant-based materials is essential to increasing their added value and expanding their industrial applications [1]. Among these resources, *Aloe vera* (*Aloe barbadensis* Miller) has attracted considerable attention due to its rich composition in polysaccharides, phenolic compounds, and other bioactive constituents, which confer important technological and biological functionalities [2].

*Aloe vera* gel is obtained from the parenchymatous tissue of the leaves and is widely recognized for its potential use in the food, pharmaceutical, and cosmetic industries [3]. Its techno-functional properties, including water retention, swelling capacity, and fat adsorption, are mainly associated with its polysaccharide matrix, particularly acemannan. Additionally, the presence of phenolic compounds, including anthraquinones and phenolic acids, contributes significantly to its antioxidant and biological activity [3,4]. However, the effective utilization of *Aloe vera* as a high-value ingredient is often limited by the sensitivity of these compounds to processing conditions, which may alter both structural integrity and functional performance [3,4].

Conventional processing methods can induce undesirable changes in the physicochemical and functional properties of *Aloe vera*, degrading bioactive compounds and reducing technological performance [3]. Therefore, there is a growing interest in the application of emerging, non-thermal technologies that enable controlled modification of plant matrices while preserving or enhancing their functional attributes. Among these technologies, high-intensity ultrasound (HIUS) has emerged as a promising non-thermal processing alternative due to its ability to induce acoustic cavitation, generating localized mechanical and physicochemical effects that can disrupt cell structures, enhance mass transfer, and modify biopolymer organization [5,6]. However, its efficiency and practical sustainability depend on the plant matrix, target compounds, acoustic conditions, energy input, equipment configuration, and processing scale [7,8].

Previous studies have demonstrated that ultrasound-assisted processing can improve selected techno-functional properties of plant-based materials, including hydration capacity and structural stability, and may favor the release or modulation of specific bioactive compounds [6,9,10]. Nevertheless, HIUS should not be considered an inherently superior extraction method, since its effect is highly matrix- and compound-dependent. In *Aloe vera*, ultrasound has been primarily studied regarding its effects on polysaccharide structure and associated functional properties [9]. However, the impact of HIUS on phenolic compounds and their contribution to antioxidant functionality remains insufficiently explored. More importantly, the relationship between cavitation-induced structural modifications and the release or degradation of bioactive compounds has not been clearly established, limiting the rational design of ultrasound-assisted processes for *Aloe vera* valorization.

In this context, understanding how processing conditions influence the structure–function–bioactivity relationship is essential in developing strategies that maximize the value of *Aloe vera* as an agro-food resource. The ability to tailor functional properties and bioactive compound availability through controlled processing would enable the design of novel ingredients with enhanced performance and application potential.

Therefore, this study aims to evaluate the effects of high-intensity ultrasound on the techno-functional, rheological, and antioxidant properties of *Aloe vera* gel, with particular emphasis on the modulation of phenolic compounds. It is hypothesized that moderate ultrasound intensities promote controlled structural disruption, enhancing the release or exposure of selected bioactive compounds and improving specific functional attributes, whereas excessive cavitation leads to degradation and loss of functionality. This work provides pilot-level evidence on the potential of HIUS for *Aloe vera* gel processing and supports future studies focused on compound-specific optimization, comparative extraction performance, energy evaluation, and scale-up assessment.

## 2. Materials and Methods

### 2.1. Raw Material

*Aloe vera* leaves, used as a raw material, were supplied by AMB Wellness Company (Gomez Palacio, Durango, Mexico). Three-year-old leaves were selected based on size uniformity. Prior to gel extraction, the leaves were washed with tap water. The *Aloe vera* gel was manually extracted as described by González-Delgado et al. [11], homogenized, and stored at 4 °C for 8 h prior to processing.

### 2.2. High-Intensity Ultrasound (US) Processing

The *Aloe vera* gel was treated acoustically using a Branson Sonifier S-450 (Branson, Danbury, CT, USA), operating at 450 W and 25 kHz, equipped with a ½-inch tip horn. Approximately 250 g of *Aloe vera* gel was placed in a double jacket vessel (400 mL). The US treatments were carried out using 3 different acoustic intensities (11, 28, and 43 W/cm^2^) for three different periods (2.5, 5, and 7.5 min) with continuous irradiation; these three acoustic intensities were chosen to represent low, intermediate, and high HIUS processing regimes, respectively. This range was established to evaluate the response of *Aloe vera* gel under different cavitation conditions, from mild ultrasound-induced effects to more intense acoustic disruption. Therefore, the selected intensities assessed whether HIUS produces gradual or threshold-dependent changes in the techno-functional, rheological, and bioactive properties of *Aloe vera* gel. Sample temperatures were controlled by recirculating water at 20 ± 2 °C. The sonicated *Aloe vera* gel was freeze-dried and stored under anhydrous conditions until analysis.

### 2.3. Techno-Functional Properties

Swelling capacity (Sw) and water retention capacity (WRC), the principal hydration-related properties, together with fat adsorption capacity (FAC), were evaluated on alcohol-insoluble residues (AIRs). Thus, AIRs from HIUS-treated and untreated *Aloe vera* gel were prepared through immersion in boiling ethanol [9]. Sw was determined as the bed volume occupied by the AIR after equilibration in an excess of solvent. Briefly, AIR samples (0.01–0.10 g) were weighed (*m*) in graduated conical tubes and mixed with an excess of phosphate buffer. The suspensions were homogenized via stirring and allowed to equilibrate for 16 h at room temperature. After equilibration, the final volume occupied by the hydrated material was recorded (*V_f_*), and Sw was expressed as mL/g AIR (Equation (1)).

(1) $$Sw=\frac{V_{f}}{m}$$

WRC was determined as the amount of water retained by the AIR after centrifugation. AIR samples (0.01–0.10 g) were suspended in phosphate buffer (5 mL) and allowed to hydrate for 24 h at room temperature. The hydrated suspensions were then centrifuged at 18,000× *g* for 15 min. Residual solids present in the supernatant were recovered via filtration using GF/C filter paper and recombined with the pellet. The hydrated pellet was weighed (*P*_1_) and subsequently dried at 102 °C overnight. After cooling, the dry weight was recorded (*P*_2_). WRC was calculated according to the following equation [9]:

(2) $$WRC=\;\frac{\left(P_{1}-P_{2}\right)}{P_{2}-k}$$

where *k* = α(*P*_1_ — *P*_2_), and α = 0.028 g phosphate/mL. WRC values were expressed as g H_2_O/g AIR.

FAC was defined as the amount of oil retained by the AIR after centrifugation. AIR samples (0.01–0.10 g; *P*_0_) were mixed with sunflower oil (5 mL) and left overnight at room temperature to enable oil adsorption. The mixtures were then centrifuged at 18,000× *g* for 10 min, the excess supernatant was carefully decanted, and the oil retained by the AIR was quantified gravimetrically (*P_f_*). FAC was expressed as g oil/g AIR (Equation (3)).

(3) $$FAC=\frac{P_{f}}{P_{0}}$$

### 2.4. Flow Behavior Analysis

Rheological measurements were performed using a controlled-stress rheometer (AR-2000, TA Instruments, New Castle, DE, USA) equipped with a parallel-plate geometry (60 mm diameter) and a Peltier plate system for precise temperature control. Sample temperature was maintained at 25 °C through water recirculation using an external thermostatic unit (Haake, Germany).

The flow behavior of untreated and HIUS-treated *Aloe vera* gel was evaluated under steady shear conditions using simple shear flow tests. Shear rate varied logarithmically from 0.01 to 300 1/s, and the corresponding shear stress values were recorded once steady-state conditions were achieved. Experimental data were fitted to different rheological models, including the Ostwald–de Waele (power law) (Equation (4)) and Cross (Equation (5)) models. All model parameters were estimated via non-linear regression analysis [12].

(4) $$\eta =K\cdot {\dot{\gamma }}^{n-1}$$

where *η* is the viscosity (Pa·s); $\dot{\gamma }$ is the shear rate (1/s); and *K* and *n* are the flow consistency ((Pa·s)*^n^*) and the flow behavior (dimensionless) indexes, respectively.

(5) $$\frac{\eta -\eta_{\infty }}{\eta_{0}-\eta_{\infty }}=\frac{1}{1+{\left(\dot{\gamma }\cdot \lambda \right)}^{m}}$$

where *η* is the viscosity at shear stable state (Pa·s); λ the structural relaxation time (s); m is the dimensionless exponent related to the shear-thinning behavior; $\dot{\gamma }$ the shear rate (1/s); and *η_∞_* and *η*_0_ the limit viscosities at high and low shear rates (Pa·s), respectively.

The goodness of fit for each model was assessed using the coefficient of determination (R^2^) and the root mean square error (RMSE). R^2^ was calculated to quantify the proportion of variance in the experimental data explained by the model, while RMSE was used as an absolute measure of the deviation between experimental and predicted shear stress values. Models presenting higher R^2^ values and lower RMSE were considered to provide a more accurate description of the flow behavior of the *Aloe vera* gel under the evaluated ultrasound processing conditions.

### 2.5. Analysis of Phenolic Compounds

#### 2.5.1. Extraction of Phenolic Compounds

The phenolic compounds were extracted as previously described by Comas-Serra et al. [13], with slight modifications. Approximately 500 mg of the freeze-dried sample was homogenized in 10 mL of H_2_O (HPLC grade). Then, the samples were centrifuged at 6000 rpm for 20 min at 25 °C. The supernatant was passed through a Ø 5 μm filter prior to spectrophotometric and HPLC analysis.

#### 2.5.2. Total Phenolic Compounds Determination

Total soluble polyphenols were spectrophotometrically measured in accordance with the Folin–Ciocalteu method, using 96-well microplates, as previously described by Comas-Serra et al. [13]. Gallic acid (0–200 ppm) was the standard for calibration, and the phenolic content results were expressed as mg of gallic acid equivalent per g of dry matter (mg GAE/g dm). Each value is the mean of six experimental determinations.

#### 2.5.3. Identification of Individual Phenolic Compounds and Aloin Using HPLC-DAD

The individual phenolic compounds, including aloin, were analyzed by HPLC-DAD according to the method described by González-Delgado et al. [11], with slight modifications. The chromatographic analysis was carried out using an HPLC Agilent 1200 (Agilent Technology, Palo Alto, CA, USA) equipped with a diode array detector (DAD), a quaternary pump, and a Kinetex C18 5-μm (250 mm × 4.6 mm) column. The temperature, flow rate, and injection loop were 25 °C, 0.5 mL/min, and 20 μL, respectively. The mobile phase comprised (A) 50 mM ammonium diacid phosphate solution brought to 2.6 pH with phosphoric acid, (B) 80% acetonitrile and 20% phase A, and (C) 200 mM phosphoric acid. The mobile phase gradient was 100% A at 5 min, 92% A and 8% B at 8 min, 14% B and 86% C at 20 min, 16.5% B and 83.5% C at 25 min, 21.5% B and 78.5% C at 35 min, 50% B and 50% C at 70 min, 100% A at 75 min, and 100% A at 80 min. The individual phenolic compounds were monitored at four wavelengths: 254, 280, 316, and 365 nm. Thirteen high-purity standards were used for the identification and quantification of the individual phenolic compounds: (1) gallic acid, (2) chlorogenic acid, (3) vanillic acid, (4) syringic acid, (5) 2,3-dihydroxybenzoic acid, (6) ferulic acid, (7) (+) catechin, (8) (-) catechin, (9) epicatechin, (10) rutin, (11) quercetin, (12) myricetin and (13) aloin.

### 2.6. Antioxidant Activity

The samples used to determine the antioxidant activity were prepared using approximately 50 mg of lyophilized *Aloe vera* juice suspended in 10 mL of distilled water. The prepared samples were continuously mixed under refrigeration (4 °C) conditions for 18 h prior to analysis. A Multiskan FC spectrophotometer (Thermo Fisher Scientific, Waltham, MA, USA) and 96-well plates were used for antioxidant activity determinations.

#### 2.6.1. Radical Scavenging DPPH Assay

The radical scavenging capacity of the *Aloe vera* juice treated with high-intensity ultrasound, measured by the DPPH radical assay, was determined as described previously [13].

#### 2.6.2. ORAC Assay

An ORAC assay of the extracts was conducted according to Ou et al. [14], using AAPH as a peroxyl radical generator, Trolox as a standard, and fluorescein as a fluorescent probe. A diluted sample (25 μL), a blank, or Trolox calibration solutions (0 to 100 μM) were mixed with 150 μL of fluorescein (8.185 × 10^−5^ mM). The plate was incubated at 37 °C for at least 30 min in a Synergy HT Multi-Detection Microplate Reader (Bio-Tek, Winooski, VT, USA). The reaction was initiated with the addition of 25 μL of AAPH reagent and shaken for 10 s at maximum intensity. Filters were used to select an excitation wavelength of 493 nm and an emission wavelength of 515 nm. The fluorescence was measured every minute for 2 h. All samples were analyzed in duplicate. The final ORAC values were calculated using area under the decay curves and expressed as μM TE/g.

### 2.7. Statistical Analysis

For the experimental responses, one-way ANOVA was applied to evaluate the effect of the HIUS treatment conditions. Fisher’s LSD test (α = 0.05) was subsequently used to identify significant differences among treatments. Rheological behavior was analyzed independently by fitting the experimental flow curves to the Ostwald–de Waele and Cross models; the corresponding model parameters were subsequently compared statistically. All statistical analyses were performed in MINITAB version 22.4.0, while the graphics were prepared using SIGMAPLOT 10.0. Note that the present study was carried out at the laboratory scale and designed as a comparative assessment of selected HIUS intensity–time combinations rather than a response surface optimization study.

## 3. Results and Discussion

### 3.1. Techno-Functional Properties

**Figure 1:**
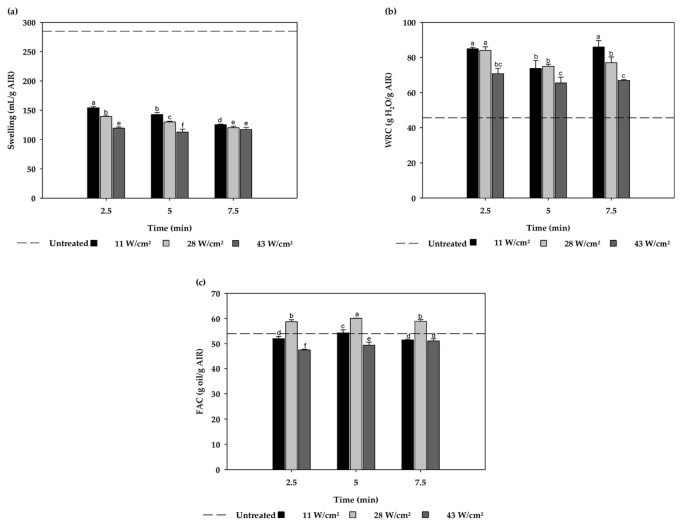
Techno-functional properties for high-intensity ultrasound (HIUS)-treated *Aloe vera* gel processed at different ultrasound intensities (11, 28, and 43 W/cm^2^) and treatment times: 2.5 min, 5 min, and 7.5 min: (**a**) swelling, (**b**) water retention, and (**c**) fat adsorption capacity. Values are presented as mean ± standard deviation. Different letters indicate statistically significant differences among treatments (*p* < 0.05).

HIUS induced significant changes (*p* < 0.05) in the techno-functional properties of *Aloe vera* gel, including Sw, WRC, and FAC (Figure 1). Since no direct structural analysis was performed, these changes were interpreted as indirect evidence of ultrasound-induced modifications in the polysaccharide-rich gel matrix. These effects may be associated with cavitation-driven disruption, partial depolymerization, molecular rearrangement, and changes in the availability of hydrophilic and hydrophobic interaction sites [15,16].

The untreated sample exhibited a swelling capacity of 284.92 mL/g AIR, whereas all HIUS-treated samples showed significantly lower values, with reductions of up to ~60% depending on processing conditions. This decrease was more pronounced at higher ultrasound intensities and longer processing times, indicating that cavitation disrupts the polymeric network responsible for water uptake. Mechanistically, the collapse in swelling capacity could be due to chain scission, reduced molecular weight, and loss of structural integrity in acemannan-rich domains [9,16]. While moderate US intensity (11 W/cm^2^) induced limited structural relaxation, preserving partial hydration capacity, excessive acoustic energy (43 W/cm^2^) led to compact and fragmented structures with minimal swelling ability.

In contrast, WRC increased significantly in response to ultrasound treatment, reaching values up to 86 ± 3.7 g/g AIR compared with 45.62 g/g AIR in the untreated sample. Moderate ultrasound conditions (11–28 W/cm^2^) produced the highest WRC values, corresponding to ~1.7–2.5-fold increases. This behavior suggests that controlled cavitation enhances the formation of a hydrated network with improved water-binding sites, likely due to partial de-esterification and increased exposure of hydrophilic groups. However, excessive ultrasound intensity reduced WRC, indicating that over-fragmentation compromises the three-dimensional structure required for effective water immobilization [9,16,17].

FAC showed a clear dependence on the ultrasound conditions, with the highest lipid-binding capacity observed at 28 W/cm^2^, particularly after 5 min, where FAC reached 60.1 ± 0.1 g/g AIR. Those treatments performed at 28 W/cm^2^ were the only ones that consistently exceeded the untreated sample (57 g/g AIR), indicating that moderate acoustic intensity favored the exposure of hydrophobic regions and increased the availability of lipid-binding sites [9,17]. In contrast, samples treated at 11 W/cm^2^ showed FAC values close to or slightly below those of the untreated gel, suggesting that this intensity was insufficient to promote major structural changes. At the highest intensity (43 W/cm^2^), FAC decreased markedly, especially at 2.5 and 5 min, which may reflect excessive cavitation effects, polymer chain disruption, or loss of organized structures required for efficient lipid retention [9]. Therefore, the figure suggests that moderate HIUS intensity improves FAC, whereas low intensity has limited effects and high intensity negatively affects lipid-binding capacity. These results demonstrate that HIUS modifies techno-functional properties through a balance between structural disruption and reorganization. Moderate ultrasound intensities promote optimal microstructural conditions that enhance hydration and lipid-binding properties, whereas excessive cavitation leads to degradation and loss of functionality. This behavior is consistent with previous reports indicating that ultrasound can modify the functional properties of *Aloe vera* through changes in its polysaccharide-rich matrix. Alvarado-Morales et al. [9] reported that thermosonication affected the functional properties and main bioactive polysaccharides of *Aloe vera* juice, with acemannan being particularly relevant for the hydration behavior of the matrix. In the present study, the decrease in Sw and the simultaneous increase in WRC under moderate HIUS conditions suggest that acoustic cavitation modified the ability of the AIR fraction to hydrate and immobilize water. This agrees with recent evidence showing that ultrasonic processing can induce chain scission and structural changes in acemannan [16]. Moreover, the higher FAC observed at 28 W/cm^2^ suggests that intermediate cavitation may expose hydrophobic interaction sites without completely disrupting the polysaccharide network. This agrees with Chokboribal et al. [17], who reported that changes in the acetylation pattern of acemannan markedly affect its physical properties and bioactivity. Therefore, compared with previous *Aloe vera* studies, the present results indicate that HIUS produces a condition-dependent response, in which moderate acoustic intensity improves specific functional attributes, whereas excessive cavitation reduces the structural organization required for swelling and lipid retention.

### 3.2. Flow Behavior

**Figure 2:**
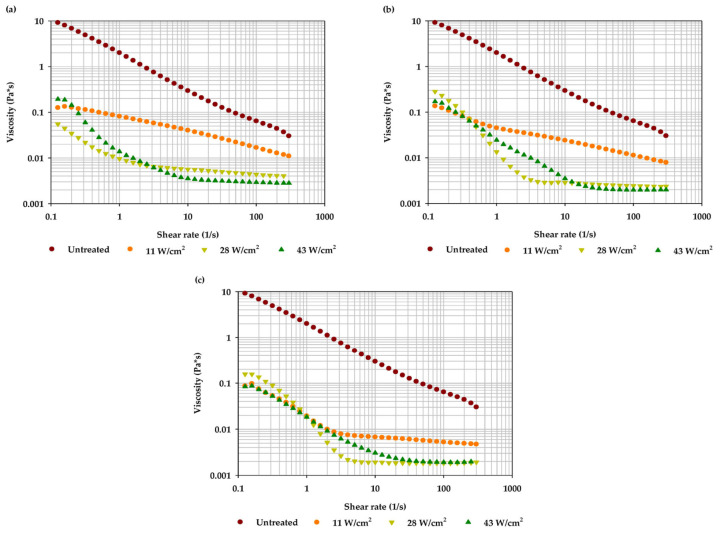
Apparent viscosity as a function of shear rate for untreated and high-intensity ultrasound (HIUS)-treated *Aloe vera* gel processed at different ultrasound intensities (11, 28, and 43 W/cm^2^) and treatment times: (**a**) 2.5 min, (**b**) 5 min, and (**c**) 7.5 min.

Figure 2 shows apparent viscosity as a function of shear rate for untreated and HIUS-treated *Aloe vera* gel processed at different ultrasound intensities (11, 28, and 43 W/cm^2^) and treatment times ((a) 2.5 min, (b) 5 min, and (c) 7.5 min). The rheological behavior of the gel was strongly affected by HIUS, providing further insight into the structural modifications induced by cavitation. All samples exhibited non-Newtonian shear-thinning behavior, characteristic of polysaccharide-based systems [18,19]. This rheological response is consistent with recent studies describing *Aloe vera* gel as a soft-textured hydrocolloid whose viscosity is primarily governed by its hydrated polysaccharide matrix. Under increasing shear rates, the alignment of polymer chains and the progressive disruption of weak intermolecular interactions reduce resistance to flow, decreasing the apparent viscosity [18].

The untreated gel showed the highest apparent viscosity, reflecting an intact and highly entangled polymer network. In contrast, ultrasound-treated samples exhibited a marked reduction in viscosity, particularly at low shear rates, indicating disruption of the gel structure [20]. At short processing times (2.5 min), viscosity decreased significantly at intermediate and high intensities (28 and 43 W/cm^2^), suggesting rapid depolymerization and chain disentanglement.

Interestingly, at the intermediate processing time (5 min), samples treated at 11 and 28 W/cm^2^ exhibited partial viscosity recovery, particularly in the low-to-intermediate shear rate range. This behavior suggests that moderate cavitation not only induces depolymerization but also promotes molecular rearrangement and re-entanglement, leading to the formation of a transient network structure [5]. In contrast, samples treated at 43 W/cm^2^ continued to exhibit low viscosity, indicating that excessive acoustic energy prevents structural reorganization.

At longer processing times (7.5 min), the divergence in rheological behavior became more evident. Low-intensity treatment (11 W/cm^2^) resulted in relatively high viscosity values, likely due to the formation of compact aggregates or shear-resistant domains [5]. Conversely, higher intensities led to extensive structural degradation and minimal resistance to flow.

The rheological behavior of untreated and HIUS-treated *Aloe vera* gel was successfully described using the Ostwald–de Waele (power law) and Cross models (Table 1). The untreated gel exhibited clear non-Newtonian, shear-thinning behavior, as evidenced by a flow behavior index (*n*) of 0.273 and a consistency index (*K*) of 0.483 Pa·s*^n^*. The low *n* value confirms the pseudoplastic nature of native *Aloe vera* gel, which is commonly associated with the presence of acemannan-rich, high-molecular-weight polysaccharides, whose acetylation pattern and interaction with pectic/cell wall polysaccharides contribute to a weak network formation. Therefore, the marked decrease in viscosity observed after HIUS treatment may reflect cavitation-induced disentanglement, partial chain scission, and weakened polysaccharide associations [12,19,20].

For the HIUS-treated samples, the Cross model provided a more comprehensive description of the flow behavior, depicting both low- and high-shear viscosity plateaus [21,22]. The zero-shear viscosity (η_0_) showed a strong dependence on ultrasound intensity and treatment time. At short treatment times (2.5 min), η_0_ decreased markedly at 11 and 28 W/cm^2^ (0.147 and 0.096 Pa·s, respectively), indicating initial disruption of the polysaccharide network due to cavitation-induced chain scission [5,16]. In contrast, treatment at 43 W/cm^2^ resulted in a higher η_0_ (0.240 Pa·s), suggesting partial structural reorganization or aggregation at higher acoustic energy. At 5 min, η_0_ increased substantially at 11 and 28 W/cm^2^, reaching 0.391 and 0.566 Pa·s, respectively, which could be due to enhanced intermolecular interactions and the re-entanglement of fragmented polysaccharide chains [15,23,24]. However, further increases in ultrasound intensity or exposure time led to a decrease in η_0_, which was particularly evident at 7.5 min and 43 W/cm^2^ (η_0_ = 0.055 Pa·s), consistent with excessive degradation of the gel network. The infinite-shear viscosity (η_∞_) remained low for most treatments (~0.002–0.013 Pa·s), indicating that, regardless of processing conditions, the gel structure was largely disrupted under high shear rates. An exception was observed for the sample treated at 11 W/cm^2^ for 7.5 min, which exhibited a markedly higher η_∞_ (0.447 Pa·s), suggesting the presence of residual or restructured aggregates resistant to shear. The characteristic relaxation time (λ) and the dimensionless parameter *m* further reflected the complex structural evolution induced by HIUS. Particularly at intermediate conditions, such as 25.727 s at 11 W/cm^2^ for 5 min, higher λ values indicate slower structural relaxation and more pronounced viscoelastic behavior. Conversely, lower λ values at higher intensities and longer treatment times suggest faster breakdown and reduced structural integrity. Variations in *m* (0.665–2.904) highlight differences in the sharpness of the transition between Newtonian plateaus, reinforcing the sensitivity of gel microstructure to ultrasound processing conditions. These results agree with previous studies describing *Aloe vera* gel and mucilage as pseudoplastic systems whose flow behavior is governed mainly by the concentration, molecular weight, acetylation degree, and organization of polysaccharides. Minjares-Fuentes et al. [12] reported that the rheological properties of *Aloe vera* mucilage are strongly associated with the composition and integrity of its water-soluble polysaccharides. Similarly, Medina-Torres et al. [20] described the shear-thinning behavior of mucilage-based systems as a consequence of polymer chain orientation and progressive disruption of weakly associated networks under shear. In the present study, the decrease in viscosity after HIUS treatment agrees with the expected effect of cavitation on polysaccharide chains, where mechanical shear and localized pressure gradients promote chain disentanglement and partial depolymerization. This is concomitant with the findings of Fu et al. [15], who reported that ultrasonic degradation of polysaccharides reduces molecular size and modifies functional behavior. However, the partial recovery of zero-shear viscosity at 11 and 28 W/cm^2^ after 5 min suggests that the response was not solely degradative but may also involve the molecular rearrangement or re-entanglement of fragmented chains. This dual response is consistent with the general behavior of hydrocolloid systems, where viscosity reflects the balance between polymer degradation, intermolecular interactions, and network reorganization [22,23,24].

### 3.3. Total Phenolic Compounds

**Figure 3:**
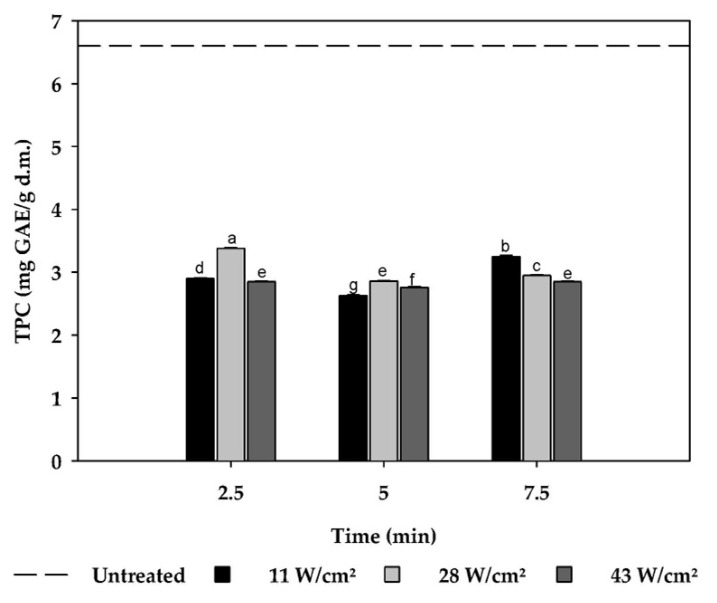
Effect of high-intensity ultrasound (HIUS: 11, 28, and 43 W/cm^2^) and processing time (2.5, 5, and 7.5 min) on total phenolic content (TPC) of *Aloe vera* gel, expressed as mg gallic acid equivalents (GAE) per g d.m. Values are presented as mean ± standard deviation. Different letters indicate statistically significant differences among treatments (*p* < 0.05).

Figure 3 shows the effect of HIUS on the total phenolic content (TPC) of *Aloe vera* gel. The unprocessed gel showed a TPC of approximately 6 mg GAE/g d.m., while the ultrasound-treated samples showed lower values, ranging from 2.63 ± 0.01 to 3.38 ± 0.01 mg GAE/g d.m., depending on the acoustic intensity and processing time. Under the evaluated conditions, these results indicate that HIUS significantly reduced TPC compared with the untreated sample (*p* < 0.05).

Among the sonicated samples, TPC varied according to both acoustic intensity and processing time. The highest value was observed at 28 W/cm^2^ for 2.5 min, suggesting that moderate intensity combined with short exposure was less detrimental to phenolic compounds than longer treatments or higher intensities. Although ultrasound may facilitate the release of phenolics from the *Aloe vera* gel matrix [10,25,26], this effect was not sufficient to compensate for their possible degradation, oxidation, or structural transformation during processing. Consequently, all HIUS-treated samples showed lower TPC than the unprocessed gel.

Note that increasing HIUS severity did not improve TPC. In general, longer processing times and higher acoustic intensities maintained or further reduced the phenolic content. This behavior suggests that the phenolic fraction of *Aloe vera* gel is sensitive to excessive sonication. Therefore, HIUS should not be interpreted only as an extraction-enhancing technology but also as a process that must be carefully optimized to balance matrix disruption and bioactive compound preservation.

The reduction in TPC after HIUS treatment may be associated with the physicochemical effects generated by acoustic cavitation [27]. The collapse of cavitation bubbles can produce localized hot spots, high-pressure gradients, and reactive free radicals, which may oxidize or transform phenolic molecules [26,27,28]. Thus, the observed TPC reduction likely reflects the interaction of two simultaneous phenomena: the release of bound or entrapped phenolics and their partial degradation during sonication. The reduction in TPC observed after HIUS treatment contrasts with several ultrasound-assisted extraction studies in which moderate acoustic energy enhances phenolic recovery from plant matrices. For instance, Comas-Serra et al. [10] reported that ultrasound-assisted aqueous extraction improved the antioxidant properties of polysaccharide-rich extracts obtained from grape stem by-products, while Gil-Martín et al. [25] emphasized that the extraction method strongly influences phenolic recovery from food industry by-products. Similarly, Korobiichuk et al. [26] and Nipornram et al. [27] described ultrasound as an efficient technology for improving mass transfer and phenolic extraction from plant tissues. However, these results indicate that *Aloe vera* gel behaves differently under the evaluated HIUS conditions, since the release of phenolics was not sufficient to compensate for their degradation or transformation. This discrepancy may be explained by differences in matrix composition, water content, polysaccharide organization, and the sensitivity of *Aloe vera* phenolics and anthraquinones to cavitation-derived reactive species. In this regard, Wang et al. [29,30] and Wang et al. [28] also reported that ultrasound-induced changes in phenolics and antioxidant activity are strongly dependent on treatment severity and matrix-specific susceptibility. Therefore, the present study demonstrates that HIUS should not be interpreted only as an extraction-enhancing process, but rather as a technology requiring precise control to avoid degradation of labile bioactive compounds.

### 3.4. Phenolic Compounds Identified by HPLC-DAD

**Figure 4:**
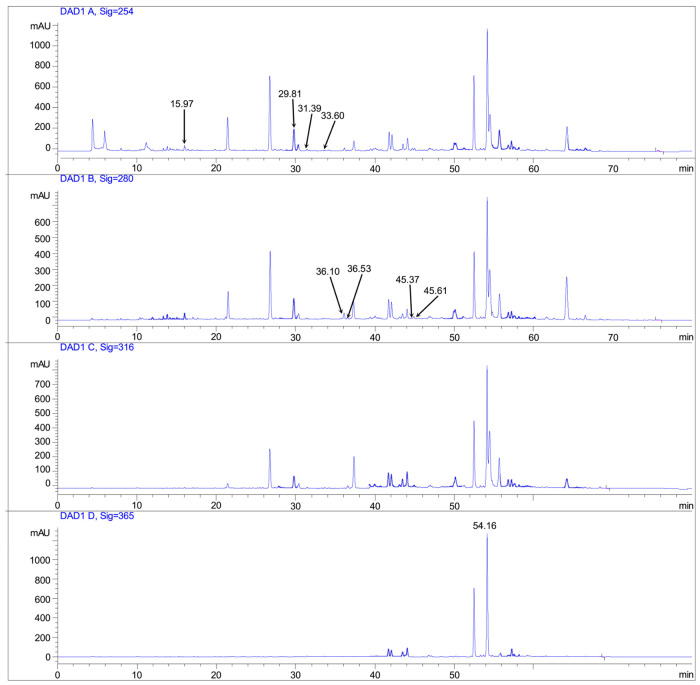
HPLC-DAD chromatogram of *Aloe vera* gel processed at 28 W/cm^2^ for 5 min: gallic acid (15.97 min); chlorogenic acid (29.81 min); vanillic acid (31.39 min); syringic acid (33.60 min); 2,3-dihydroxibenzoic acid (36.10 min); epicatechin (36.53 min), ferulic acid (45.37 min); rutin (45.61 min); aloin (54.16 min).

The analysis of individual phenolic compounds by HPLC-DAD revealed compound-specific responses to ultrasound treatment (Figure 4). Table 2 summarizes the effect of US processing intensity (11, 28, and 43 W/cm^2^) and treatment time (2.5, 5.0, and 7.5 min) on the concentration of individual phenolic compounds. At the shortest treatment time (2.5 min), increasing US intensity generally promoted higher phenolic release. In particular, gallic acid exhibited a strong intensity dependence, increasing from 1.91 μg/mg d.m. at 11 W/cm^2^ to 5.75 μg/mg d.m. at 43 W/cm^2^. A similar trend was observed for 2,3-dihydroxybenzoic acid and ferulic acid, whose concentrations reached their highest values at 43 W/cm^2^. In contrast, syringic acid and rutin were not detected at low intensity but instead became detectable at higher US intensities, indicating ultrasound-assisted liberation from the plant matrix. At the intermediate processing time (5 min), most phenolic acids showed maximum or near-maximum concentrations at 28 W/cm^2^, suggesting an optimal cavitation regime for compound release. Gallic and vanillic acids increased relative to 2.5 min treatments at low and moderate intensities, whereas excessive intensity (43 W/cm^2^) resulted in reduced gallic acid concentration, suggesting partial degradation or transformation under prolonged high-energy conditions. Epicatechin exhibited a pronounced decrease at 28 W/cm^2^ (0.23 μg/mg d.m.), indicating higher sensitivity to ultrasonic conditions compared with phenolic acids. At the longest processing time (7.5 min), phenolic profiles revealed divergent behaviors. Syringic, ferulic acids, epicatechin, and rutin reached their highest concentrations at 43 W/cm^2^, highlighting the role of prolonged cavitation in enhancing the release of more strongly bound or less extractable compounds. Conversely, gallic and chlorogenic acids showed relatively stable or reduced concentrations compared to intermediate conditions, suggesting that extended exposure may favor degradation or conversion reactions for these more labile phenolics. Taken together, the data demonstrate that ultrasound processing modifies the phenolic profile of *Aloe vera* in a selective and process-dependent manner, governed by the combined effects of acoustic intensity and treatment time. Under moderate-to-high ultrasonic intensities, cavitation may favor the disruption of cell wall structures and enhance the release of phenolic acids and flavonoids from the plant matrix. However, when the energy input or exposure time becomes excessive, the same cavitation-derived effects may also promote oxidation, structural transformation, or degradation of specific compounds [27,28,30]. Thus, the changes observed in the phenolic profile should be interpreted as the result of a dynamic balance between extraction enhancement and compound instability.

This result is supported by the compound-specific response observed by HPLC-DAD, which confirms that ultrasound does not affect all phenolics uniformly. While some phenolic acids increased under moderate-to-high intensities, others decreased or were not detected under specific processing conditions. This behavior is consistent with the general principle of ultrasound-assisted extraction, where release, diffusion, oxidation, and degradation of phenolic compounds may occur simultaneously [25,26,27,28]. In this context, phenolics with hydroxylated aromatic structures may be more susceptible to oxidation under cavitation-derived reactive environments, whereas compounds more strongly associated with the matrix may require higher acoustic intensity or longer exposure to be effectively released. Therefore, the divergent behavior of gallic, chlorogenic, syringic, and ferulic acids, as well as epicatechin and rutin, can be attributed not only to processing severity but also to differences in chemical structure, solubility, matrix localization, and intrinsic stability, as previously discussed by Wang et al. [29], Wang et al. [30], and Wang et al. [28]. These findings emphasize the need to optimize ultrasound parameters to maximize the recovery of the targeted phenolic compounds while minimizing their degradation.

From a target selection perspective, the present results indicate that the most relevant compounds for further HIUS optimization in *Aloe vera* are not total phenolics as a whole but selected molecules or fractions showing condition-dependent responses, including specific phenolic acids, rutin, epicatechin, and aloin. However, direct comparison with conventional extraction and other emerging technologies would be required to determine whether HIUS provides superior yield, selectivity, or process efficiency for these targets.

### 3.5. Aloin

**Figure 5:**
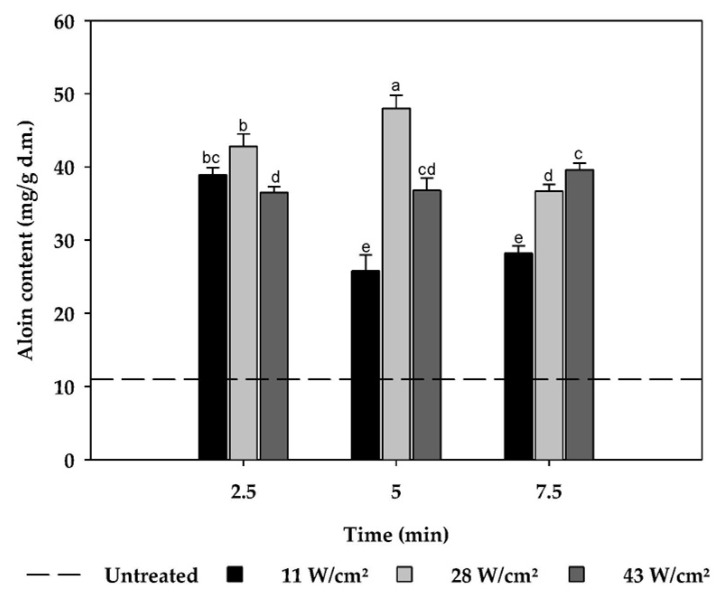
Effect of high-intensity ultrasound (HIUS: 11, 28, and 43 W/cm^2^) and processing time (2.5, 5, and 7.5 min) on aloin content in *Aloe vera* gel, expressed as mg/g d.m. Values are presented as mean ± standard deviation. Different letters indicate statistically significant differences among treatments (*p* < 0.05).

The concentration of aloin present in the different Aloe samples treated with ultrasound is shown in Figure 5. Applying high-intensity US promoted significant changes in the aloin content, and this exhibited a non-linear response to ultrasound processing, reinforcing the concept of a dynamic balance between compound release and degradation. At low intensity (11 W/cm^2^), aloin decreased with increasing processing time, indicating progressive degradation. This behavior can be attributed to prolonged cavitation exposure leading to structural degradation or transformation of aloin, an anthrone C-glycoside known to be susceptible to hydrolysis and oxidation under energetic conditions. Previous studies indicate that anthraquinones and related glycosides can undergo conversion to aloe emodin or other derivatives when subjected to intense physicochemical stress, including thermal, acidic, or mechanical treatments [2,31]. Although ultrasound is considered a non-thermal technology, localized hotspots and free radical generation associated with acoustic cavitation may promote similar degradation pathways during extended processing times. At intermediate intensity (28 W/cm^2^), aloin concentration increased significantly at 5 min, followed by a decrease at 7.5 min, reflecting an optimal extraction range. The increase in aloin content observed at 5 min could be due to the improved aloin release from the latex-containing tissues as a consequence of enhanced cell wall disruption and mass transfer promoted by the ultrasound [26,32]. This phenomenon aligns with previous reports showing that moderate intensities of emerging technologies facilitate the liberation of phenolic compounds by disrupting lignocellulosic matrices and vascular bundles where anthraquinones are predominantly localized [33,34]. Conversely, the marked reduction observed at 7.5 min aligns with the idea of phenolic compounds, including anthraquinones, in *Aloe vera* and other plant matrices subjected to intensified processing, where excessive energy input results in phenolic breakdown rather than further release [33].

At high intensity (43 W/cm^2^), aloin content remained relatively constant, suggesting that release and degradation processes occurred simultaneously. Given the known instability and regulatory implications of aloin, these findings highlight the importance of controlling ultrasound conditions to tailor its concentration in *Aloe vera*-derived products. Anthraquinones, including aloin, are characteristic bioactive constituents of *Aloe vera*; however, their limited stability under severe processing conditions and the regulatory concerns associated with their presence in food and nutraceutical products highlight the need for careful process control [2,33,34].

The non-linear behavior observed in this study supports the dual role of high-intensity ultrasound as a technology capable of enhancing mass transfer and bioactive release under short or moderate treatments, while also promoting degradation or transformation reactions under prolonged exposure. This is consistent with Gansukh et al. [35], who reported that 5 min of ultrasound-assisted extraction improved the recovery of aloin and aloe emodin from *Aloe vera* extracts, supporting the ability of short sonication treatments to favor the release of anthraquinone derivatives. Accordingly, the increase in aloin at 28 W/cm^2^ for 5 min can be attributed to an extraction-enhancement effect, whereas the subsequent decrease after 7.5 min suggests a shift toward compound instability. Similar considerations have been reported for *Aloe vera* secondary metabolites, whose recovery, preservation, and bioactivity are strongly influenced by extraction and processing conditions [2,31,33,34]. Therefore, the HIUS should not be considered as exerting a unidirectional effect on aloin content, but rather as a process-dependent tool capable of modulating its concentration within a limited set of processing conditions in which release predominates over degradation. Optimization of ultrasound intensity and treatment time is consequently essential to tailor *Aloe vera* extracts for food applications, either by enhancing the recovery of bioactive constituents for functional ingredients or by reducing aloin levels to comply with the safety and quality requirements.

### 3.6. Antioxidant Activity and Radical Scavenging Capacity of Aloe vera

**Figure 6:**
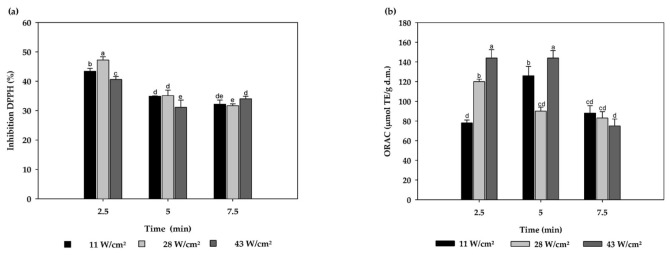
(**a**) DPPH radical scavenging activity (%) and (**b**) oxygen radical absorbance capacity (ORAC; μmol TE/g d.m.) of *Aloe vera* gel subjected to ultrasound (US) treatment at different power densities (11, 28, and 43 W/cm^2^) and processing times (2.5, 5, and 7.5 min). Results are expressed as mean ± standard deviation. Different letters denote statistically significant differences among treatments (*p* < 0.05).

The radical scavenging capacity (RSC) of the *Aloe vera* juice treated with high-intensity ultrasound is shown in Figure 6. The juice’s antioxidant response clearly depended on processing severity, as evidenced by the complementary DPPH and ORAC assays. In agreement with the trends observed for TPC and the individual phenolic profile in this study, both assays confirm that antioxidant functionality is also governed by a dynamic balance between cavitation-induced release and degradation of bioactive compounds.

DPPH radical scavenging activity (31.1 ± 2.5–47.2 ± 1.1%) reached its maximum under moderate conditions (28 W/cm^2^, 2.5 min), which correlates with the highest TPC value. This correlation supports the widely reported role of phenolic compounds as primary contributors to reducing capacity in plant matrices. Similar behavior has been described in ultrasound-treated fruit systems, where moderate acoustic energy enhances the extractability of phenolics through cell wall disruption and increased solvent penetration, resulting in improved DPPH activity [29,30,32]. In *Aloe vera*, this effect is particularly relevant due to the association of phenolics with the polysaccharide matrix, as previously reported for ultrasound-assisted extraction and processing [9,10]. However, the decrease in DPPH activity observed at longer processing times (≥5 min) indicates that extended cavitation promotes degradation pathways. This behavior is consistent with reports showing that ultrasound can generate reactive oxygen species (ROS) and localized hotspots, which accelerate oxidation and structural breakdown of phenolic compounds [2,33].

In contrast, the ORAC assay showed a broader response (75 ± 7–144 ± 8.5 µmol TE/g) and a stronger dependence on ultrasound intensity, with maximum values observed at 43 W/cm^2^, particularly at short processing times. This divergence from DPPH suggests that ORAC captures a wider spectrum of antioxidant compounds, including those acting through hydrogen atom transfer (HAT) mechanisms and exhibiting higher resistance to oxidative degradation. Similar discrepancies between DPPH and ORAC responses have been reported in plant-based systems, where ultrasound selectively enhances the release of different classes of antioxidants depending on their localization and chemical stability [34]. In the present study, the sustained ORAC values at intermediate processing times (5 min) further suggest that some antioxidant compounds are less susceptible to degradation than simple phenolic acids, potentially flavonoids, or more complex phenolics identified using HPLC-DAD.

In general, DPPH and ORAC show that HIUS does not uniformly affect antioxidant activity but rather modulates the antioxidant profile selectively. This selectivity could be due to differences in compound stability, molecular structure, and interaction with the polysaccharide matrix. Ultrasound-induced depolymerization of acemannan and related polysaccharides, as demonstrated in previous studies [9,17], likely enhances the release of bound phenolics while simultaneously exposing them to oxidative environments. Thus, the observed antioxidant response reflects not only the concentration of released compounds but also their structural integrity and reactivity.

From a mechanistic standpoint, the results align with the dual role of acoustic cavitation. At low-to-moderate processing severity, cavitation bubbles collapse and generate shear forces that disrupt cellular structures, enhancing mass transfer and the liberation of bioactive compounds [27]. This mechanism has been widely recognized as the primary driver of ultrasound-assisted extraction and functional enhancement [9,10]. However, as processing intensity or time increases, secondary effects such as ROS generation, thermal microgradients, and mechanical stress become dominant, promoting degradation reactions that reduce antioxidant capacity [2,27,28,30,33]. This transition explains the decline observed in both DPPH and ORAC at prolonged treatment times (7.5 min), despite continued structural disruption.

Notably, an optimal condition identified in this study (28 W/cm^2^, 2.5 min) is consistent with the processing range reported in ultrasound applications where moderate energy input maximizes functional properties while minimizing degradation [10,27]. This condition ensures sufficient structural modification to enhance phenolic release without exceeding the threshold at which oxidative degradation becomes significant. Similar optimal regimes have been reported in other plant matrices, highlighting the importance of carefully controlling ultrasound parameters to achieve the desired functional properties [30,33,34]. These results show that HIUS is a powerful tool for modulating the antioxidant properties of *Aloe vera*, but its effectiveness depends critically on processing conditions. The differential response observed between the DPPH and ORAC assays underscores the importance of using complementary analytical approaches to capture the complexity of antioxidant systems. The antioxidant response observed in this study also agrees with the previous reports showing that ultrasound effects on antioxidant activity are highly dependent on the balance between enhanced release and degradation of bioactive compounds. Wang et al. [30] reported that high-intensity ultrasound modified phenolics, antioxidant activity, and microstructure in strawberry juice, while Wang et al. [29] observed that ultrasound processing affected total phenolics, flavonoids, ascorbic acid, and antioxidant capacity in kiwifruit juice depending on the applied conditions. In the present study, the highest DPPH inhibition under moderate conditions agrees with the concept that controlled cavitation can improve the availability of compounds. However, the lack of direct proportionality between TPC, DPPH, and ORAc indicates that antioxidant activity was not governed by total phenolic concentration. Instead, the response likely depended on the specific phenolic profile, aloin content, degradation products, and different mechanisms involved in each antioxidant assay. This behavior is consistent with reports indicating that ultrasound can selectively affect different antioxidant compounds depending on their localization, polarity, and chemical stability [10,29,30,32]. Therefore, the present results support the existence of an optimal HIUS processing range in which the functional properties of *Aloe vera* can be enhanced while minimizing oxidative degradation and preserving the integrity of bioactive compounds, thereby contributing to the development of high-value *Aloe vera*-based ingredients for agro-food applications. However, additional technical parameters, including energy demand, acoustic efficiency, processing scale, and reactor configuration, should be considered in future studies, together with comparative assessments against non-thermal technologies or solvent systems [7,8].

## 4. Conclusions

This study shows that, under the evaluated laboratory-scale conditions, high-intensity ultrasound significantly modifies the structural, functional, and antioxidant properties of *Aloe vera* gel through cavitation-induced mechanisms. Under moderate ultrasound conditions, controlled matrix disruption and reduced viscosity favored the modulation or release of specific bioactive compounds, enhanced selected antioxidant activity, and improved water retention capacity. In contrast, excessive acoustic intensity or prolonged processing caused over-fragmentation, reducing swelling capacity, decreasing viscosity, and compromising the stability of phenolic compounds and aloin. Overall, these findings confirm that HIUS application to *Aloe vera* gel involves both beneficial and detrimental effects. These findings should be considered pilot-level evidence of the HIUS’s potential to modulate *Aloe vera* gel functionality and selected bioactive compounds, rather than as proof of generalizable extraction efficiency or environmental superiority. Identifying an optimal processing range is essential to maximizing the technological, nutritional, and application potential of *Aloe vera*-based ingredients in food systems. Therefore, although HIUS shows potential as a non-thermal strategy, its practical sustainability and industrial relevance should be confirmed through future studies addressing energy demand, acoustic efficiency, scale-up feasibility, techno-economic performance, life-cycle impact, and target-compound value.

## Figures and Tables

**Table 1:** Rheological parameters of untreated and high-intensity-ultrasound-treated *Aloe vera* gel, determined using the Ostwald–de Waele and Cross models.

		Ostwald–de Waele (Power Law) Model		Cross Model				R^2^	RMSE
		*K*	*n*	η_0_	η_∞_	λ	*m*		
		Pa·s*^n^*	-	Pa·s	Pa·s	s	-		
Untreated *Aloe vera* gel		0.483	0.273					0.9966	0.1608
High-intensity ultrasound treatment									
min	W/cm^2^								
2.5	11			0.147	0.010	0.750	0.665	0.9904	0.0038
2.5	28			0.096	0.004	7.641	1.113	0.9626	0.0023
2.5	43			0.240	0.005	5.730	2.904	0.9938	0.0043
5	11			0.391	0.013	25.727	0.736	0.9673	0.0051
5	28			0.566	0.002	9.791	1.957	0.9997	0.0009
5	43			0.388	0.002	11.870	1.240	0.9981	0.0020
7.5	11			0.569	0.447	7.514	2.053	0.9760	0.0034
7.5	28			0.137	0.003	2.072	2.768	0.9842	0.0060
7.5	43			0.055	0.002	1.291	1.288	0.9885	0.0030

**Table 2:** Concentration of phenolic compounds (μg/mg d.m.) identified by HPLC-DAD in *Aloe vera* gel subjected to ultrasound (US) treatment at different power densities (11, 28, and 43 W/cm^2^) and processing times (2.5, 5, and 7.5 min).

Time (min)	US (W/cm^2^)	Gallic Acid	Chlorogenic Acid	Vanillic Acid	Syringic Acid	2,3-Dihydroxybenzoic Acid	Ferulic Acid	Epicatechin	Rutin
2.5	11	1.91 ± 0.10 e	3.23 ± 0.19 c	0.48 ± 0.03 a	n.d.	1.19 ± 0.05 f	0.17 ± 0.08 c	1.07 ± 0.03 e	n.d.
2.5	28	2.19 ± 0.04 d	3.57 ± 0.12 b	0.52 ± 0.01 a	n.d.	1.48 ± 0.04 d	0.17 ± 0.11 c	1.33 ± 0.10 d	0.91 ± 0.12 f
2.5	43	5.75 ± 0.06 a	3.72 ± 0.23 ab	0.24 ± 0.01 b	1.05 ± 0.04 e	2.26 ± 0.12 c	0.42 ± 0.09 ab	2.02 ± 0.08 b	1.97 ± 0.02 b
5	11	2.79 ± 0.14 c	3.89 ± 0.04 a	0.10 ± 0.08 c	3.31 ± 0.02 b	2.50 ± 0.03 b	0.24 ± 0.08 bc	0.78 ± 0.08 f	1.16 ± 0.11 e
5	28	3.33 ± 0.13 b	3.67 ± 0.07 ab	0.25 ± 0.07 b	2.76 ± 0.10 d	2.61 ± 0.10 b	0.35 ± 0.12 ab	0.23 ± 0.11 h	1.62 ± 0.08 c
5	43	1.67 ± 0.03 f	2.95 ± 0.11 d	0.27 ± 0.12 b	3.18 ± 0.03 c	n.d.	0.39 ± 0.15 ab	1.77 ± 0.06 c	1.38 ± 0.08 d
7.5	11	1.31 ± 0.09 g	3.12 ± 0.09 cd	0.29 ± 0.09 b	3.34 ± 0.03 b	n.d.	0.25 ± 0.17 bc	1.15 ± 0.09 e	1.87 ± 0.03 b
7.5	28	1.45 ± 0.11 g	2.95 ± 0.21 d	0.18 ± 0.09 bc	2.73 ± 0.05 d	1.35 ± 0.02 e	0.27 ± 0.09 bc	0.50 ± 0.10 g	1.68 ± 0.12 c
7.5	43	1.80 ± 0.05 e	3.90 ± 0.06 a	0.33 ± 0.01 b	5.00 ± 0.06 a	2.84 ± 0.08 a	0.49 ± 0.09 a	2.55 ± 0.08 a	3.18 ± 0.05 a

Results are expressed as mean ± standard deviation (n = 3) in scaled units, as indicated for each compound. Different letters within the same column indicate statistically significant differences among treatments (*p* < 0.05). n.d.: not detected.

## Data Availability

The original contributions presented in this study are included in the article. Further inquiries can be directed to the corresponding author.
